# Heat and health in Antwerp under climate change: Projected impacts and implications for prevention

**DOI:** 10.1016/j.envint.2017.11.012

**Published:** 2018-02

**Authors:** Gerardo Sanchez Martinez, Julio Diaz, Hans Hooyberghs, Dirk Lauwaet, Koen De Ridder, Cristina Linares, Rocio Carmona, Cristina Ortiz, Vladimir Kendrovski, Raf Aerts, An Van Nieuwenhuyse, Maria Bekker-Nielsen Dunbar

**Affiliations:** a The UNEP-DTU Partnership, United Nations City, Marmorvej 41, Copenhagen, Denmark; b WHO European Centre for Environment and Health (World Health Organization Regional Office for Europe), Platz der Vereinten Nationen 1, Bonn, Germany; c National School of Public Health, Carlos III Institute of Health (ISCIII), Spain; d Flemish Institute for Technological Research (VITO), Belgium; e Health and Environment, Scientific Institute of Public Health (WIV-ISP), Belgium; f Department of Earth and Environmental Sciences, University of Leuven (KU Leuven), Belgium; g Department of Public Health and Primary Care, Environmental Health, University of Leuven (KU Leuven), Belgium; h Public Health England (PHE), United Kingdom

**Keywords:** Heatwaves, Antwerp, Heat-related mortality, Heat-related hospital admissions, Climate change

## Abstract

**Background:**

Excessive summer heat is a serious environmental health problem in several European cities. Heat-related mortality and morbidity is likely to increase under climate change scenarios without adequate prevention based on locally relevant evidence.

**Methods:**

We modelled the urban climate of Antwerp for the summer season during the period 1986–2015, and projected summer daily temperatures for two periods, one in the near (2026–2045) and one in the far future (2081–2100), under the Representative Concentration Pathway (RCP) 8.5. We then analysed the relationship between temperature and mortality, as well as with hospital admissions for the period 2009–2013, and estimated the projected mortality in the near future and far future periods under changing climate and population, assuming alternatively no acclimatization and acclimatization based on a constant threshold percentile temperature.

**Results:**

During the sample period 2009–2013 we observed an increase in daily mortality from a maximum daily temperature of 26 °C, or the 89th percentile of the maximum daily temperature series. The annual average heat-related mortality in this period was 13.4 persons (95% CI: 3.8–23.4). No effect of heat was observed in the case of hospital admissions due to cardiorespiratory causes. Under a no acclimatization scenario, annual average heat-related mortality is multiplied by a factor of 1.7 in the near future (24.1 deaths/year CI 95%: 6.78–41.94) and by a factor of 4.5 in the far future (60.38 deaths/year CI 95%: 17.00–105.11). Under a heat acclimatization scenario, mortality does not increase significantly in the near or in the far future.

**Conclusion:**

These results highlight the importance of a long-term perspective in the public health prevention of heat exposure, particularly in the context of a changing climate, and the calibration of existing prevention activities in light of locally relevant evidence.

## Highlights

• The relationship between heat and health is studied locally for Antwerp, Belgium.
• Health outcomes attributable to heat are assessed retrospectively and under RCP8.5.
• Projected population transitions and acclimatisation or its absence are modelled.
• Results show significant expected increases in mortality attributable to heat.
• Heat-health prevention should take climate change into account from the inception.

## 1 Background

The epidemiologic evidence on the association between heat and health impacts is clear and well established in major cities of western Europe (Analitis et al., 2014, D'Ippoliti et al., 2010, Leone et al., 2013, Michelozzi et al., 2009), especially concerning the relationship between high temperatures and mortality, and several of the risk factors in the relevant causal pathways (Bouchama et al., 2007, Kovats and Hajat, 2008). Baccini et al. studied the relationship between daily maximum apparent temperatures and mortality in 15 European cities, finding various degrees of increase in mortality in Mediterranean cities and north-continental cities, respectively, for every 1 °C increase in maximum apparent temperature above a city-specific threshold (Baccini et al., 2008). An additional study, conducted across 12 European cities, found a positive association between temperature and hospital admissions for respiratory disorders (Michelozzi et al., 2009). Subsequent studies have confirmed similar findings.

Heat-related health effects are likely to exacerbate in these urban settings under climate change, with projected rising temperatures, an increase in frequency and intensity of heat waves in the European Region (IPCC, 2013) and an intensification of the urban heat island effect (Founda and Santamouris, 2017, Wouters et al., 2017). In the absence of adaptation, an increase in heat-related adverse health effects may follow (Ciscar et al., 2014, Hajat et al., 2014, Petkova et al., 2014). Notwithstanding the increase in the available evidence, there are several major urban agglomerations in Europe for which no study has been published on the local links between heat and health. This hampers the ability to plan and implement adequate prevention, and evidence-based health adaptation to climate change.

Heat-related mortality has been described in Belgium from the early 1990s (Sartor et al., 1995, Sartor et al., 1997), and thereafter very strong impacts have been confirmed in 2003 (Sartor, 2004) and 2006 (Maes et al., 2007). In this paper, we examine retrospectively the association between temperature and mortality, and selected non-fatal outcomes, in the city of Antwerp, in Belgium, in the period 2009 to 2013. Thereafter, we estimate the changes in heat-related mortality under likely climate change and population scenarios in two future time periods (2026–2045 and 2081–2100), in the absence of adequate adaptation, and discuss policy implications in the context of current prevention and adaptation efforts in Belgium.

## 2 Methods

### 2.1 Current and future Antwerp urban climate assessment

The retrospective meteorological data series is based on temperatures derived by UrbClim, an urban climate model designed to model the urban influence on large-scale meteorological conditions at a resolution of a few hundred of metres (De Ridder et al., 2015, Lauwaet et al., 2015). The model solves a set of simplified prognostic flow equations for the atmospheric boundary layer and contains detailed urban surface physics, taking into account the reduced vegetation and increased soil sealing in the city centre. The synoptic (large-scale) atmospheric boundary conditions are taken from the global ERA-Interim reanalysis data set of the European Centre for Medium-Range Weather Forecasts (ECMWF) (Dee et al., 2011). Local terrain and surface data are based on open-source datasets, such as the Corine land cover and the European Environment Agency soil sealing data set for Europe. The model has previously been validated with several validation campaigns, among which one has focussed on the agglomeration of Antwerp (De Ridder et al., 2015, Garcia-Diez et al., 2016, Laaidi et al., 2011). Using the UrbClim model, daily urban climate data has been composed for all summer periods (May–September) for a climate reference period (1986–2015). The model provides gridded hourly data with a 250 m resolution for the entire urban agglomeration of Antwerp, which occupies a domain of approximately 20 by 20 km. This raw output is subsequently converted to daily minimal, mean and maximal temperature values, and for each of the 22 municipalities within the agglomeration, the (spatial) mean over the municipality is computed.

Future climate data has been compiled using a statistical method, as described in detail in (Lauwaet et al., 2015). We have composed projected summer daily minimal, maximal and mean temperatures for two periods, one in the near (2026–2045) and one in the far future (2081–2100), by rescaling reference temperatures (1986–2015) according to the monthly temperature changes observed in an ensemble of global climate models (GCMs). For the study at hand, the temperature rescaling functions are based on the output of a set of GCMs contained in the archives of the Coupled Model Intercomparison Project (CMIP5) archive of the Intergovernmental Panel on Climate Change (IPCC, 2013). Based on data requirements and availability, eleven GCMs have been selected, which have to form a representative set of the GCMs contained in the entire CMIP5 archive (Lauwaet et al., 2015). Due to the large computational demand of the study, only one climate scenario has been used. In its most recent assessment report (AR5), the IPCC has identified four pathways (Representative Concentration Pathways, RCPs) (IPCC, 2013), ranging from strong (RCP2.6) to weak mitigation (RCP8.5). To provide a range for the negative effects of climate change on human health, we have focused on the RCP8.5 scenario. Although this is the IPCC-scenario with the largest warming potential, global emission trends still track along the lines of this scenario (Peters et al., 2012).

### 2.2 Health impact assessment

The geographical area under study comprises the municipality of Antwerp (Belgium) for which the daily mortality series (all non-accidental causes, ICD-10 codes: A00-R99) was collected for the period 2009–2013. Initially, mortality data were collected for the 22 municipalities that can be loosely defined as the greater Antwerp area. However, for these surrounding municipalities, both separately and in aggregate, the mortality count was too low to find statistical associations. Therefore, the analysis included only temperature and health outcome data for the city (municipality) of Antwerp for the years 2009 to 2013, for which data were provided by the Flemish Agency of Public Health. In addition, the daily series of emergency admissions due to cardiorespiratory causes (ICD-10 codes: I00-I99 and J00-J99) were collected for the same period. Population data for the city of Antwerp were obtained from Statistics Belgium for the period 1986–2015. Projections for the periods 2026–2045 and 2081–2100 were based on the United Nations World Population Prospects (WPP) forecasts for Belgium (UNDESA, 2013). Taking as reference the population data for the city of Antwerp in the year 2015 (516.009 inhabitants), we calculated the proportion it represented from the total national population. Thereafter, that proportion was assumed constant, whereby the population of Antwerp would behave like that of Belgium. The specific WPP scenario chosen was the “medium variant” (UNDESA, 2008).

#### 2.2.1 Determination of heatwave threshold temperature

As the dependent variable, we used data on daily mortality due to natural causes in the summer months in Antwerp from 1st January 2009 to 31st December 2013. As the independent variable, we used data on the daily maximum and minimum temperatures in this city across the same period. Instead of using daily mortality data, we chose to work with mortality residuals, thereby eliminating trend and seasonalities from the mortality series, leaving anomalies in mortality to be related to temperature. The residuals were obtained by means of univariate autoregressive integrated moving average (ARIMA) modelling (Box et al., 1994). The advantage of working with residuals as opposed to daily mortality is that, once modelled, residuals display neither trends nor periodicities (both of which are inherent in daily mortality), with the result that any associations found will show a genuine temperature–mortality relationship from a statistical standpoint (with significance cut-off *p* < 0.05). We proceeded to plot the following on a scatter plot diagram: the mean value of the mortality series residuals on the same day (vertical axis); the maximum daily temperatures at 2 °C intervals (horizontal axis), and their corresponding 95% confidence intervals (CIs). When these mortality residuals are showed in a scatter plot with the maximum temperature data, the deviations detected correspond to real mortality anomalies. The temperature from which the mortality residuals increased significantly vis-à-vis the mean would thus be the threshold temperature.

#### 2.2.2 Impact of heat on daily mortality

The impact of temperature on mortality was quantified, using generalized linear model (GLM) methodology, with the Poisson regression link. This methodology allows us to obtain the increase in the relative risk (*RR*) of morbidity and daily mortality associated with an increase in the maximum daily temperature, and subsequently calculate the attributable risk (*AR*) associated with this increase, via the following equation (Coste and Spira, 1991):

$$\mathrm{AR}=\left[\frac{\mathrm{RR}-1}{\mathrm{RR}}\right]\cdot 100.$$

On fitting the model, we controlled for seasonalities of a five-, four- and three-monthly nature, using the sine and cosine functions with these same periodicities; and for trend and the possible autoregressive nature of the series. To consider the effect of heat stress through maximum daily temperature (*T*_max_), we created the variable *T*_heat_ defined on the basis of the previously calculated mortality threshold temperatures. Given that the effect of heat stress on morbidity and mortality may not be immediate, the following lagged variables were calculated: *T*_heat_ (lag 1), which takes into account the effect of the temperature on day *d* on mortality, one day later, *d* + 1; *T*_heat_ (lag 2), which takes into account the effect of the temperature on day *d* on mortality, two days later, *d* + 2; and so on successively. The number of lags were selected on the basis of the literature, which establishes that the effect of heat is short-term (*T*_heat_: lags 1–4) (Alberdi et al., 1998).

Furthermore, given the known synergic effects between heat, particulate matter and ozone (Diaz et al., 2002, Stafoggia et al., 2008, Zmirou et al., 1998), the variables of chemical air pollution available were also introduced as control variables. Particulate matter measurements of the urban background telemetric station in Borgerhout (in the centre of the Antwerp agglomeration) have been acquired from the Flemish Environmental Agency. Based on previous studies (Jimenez et al., 2009, Linares et al., 2006), the relationship between PM_10_ and mortality is assumed to be linear, with an effect on mortality until lag 4. The corresponding lagged variables were therefore created until this lag, as in the case of temperature. To determine the significant variables in the modelling process to be used for calculating the *RR*s and *AR*s, the backward stepwise procedure was used, beginning with the model that included all the explanatory variables, and gradually eliminating those which individually displayed least statistical significance, with the process being reiterated until all the variables included were significant at *p* < 0.05. Modelling was performed for the summer months (May–September).

To calculate the mortality attributable to heat we used a well-tested methodology (Carmona et al., 2016a). Firstly, we calculated the number of degrees whereby the maximum daily temperature exceeded the threshold temperature on each day. Thereafter, having ascertained the percentage increase in mortality for each °C via the *AR*, the total mortality percentage for the overall number of degrees whereby maximum daily temperatures exceeded the threshold temperature across the period 2009–2013 would be:

$$\%\text{mortality attributable to heat}=\mathrm{AR}\cdot {\text{excess}}^{{}^{\circ}}C.$$

Hence, to go from percentage to daily mortality, it suffices to take into account the mean mortality in Antwerp during hot days, meaning days with mortality attributable to heat, as follows:

$$\text{mortality attributable to heat}=\frac{\%\text{mortality attributable to heat}\cdot \text{mean mortality}}{100}$$

$$\text{daily mortality attributable to heat}=\frac{\text{mortality attributable to heat}\;}{\text{number of hot days}}$$

All analyses were performed using the IBM SPSS Statistics v22 and STATA v11.2 statistical software programmes.

## 3 Results

### 3.1 Heat–mortality relationship for the sample period (2009–2013)

**Fig. 1:**
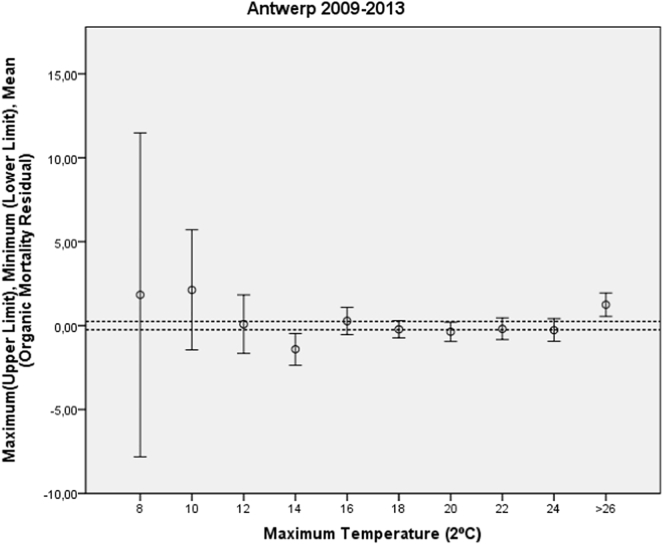
Scatter-plot diagram for determining heat-wave definition temperature on the basis of maximum daily temperature: Antwerp (2009–2013).

The mean value of the residuals of this model for every 2 °C increase in maximum daily temperature is shown on a scatter-plot diagram (Fig. 1), together with the corresponding 95% confidence intervals (CIs), and the 95% CIs of the mean of the residuals for the entire study period (represented by parallel, dashed lines).

From a maximum daily temperature of 26 °C upwards, the residual anomalies with their CIs do not coincide with the CI of the mean of the residuals for the overall period, shown as centred on zero. It is from a maximum daily temperature of 26 °C, therefore, that heatwave-related mortality can be said to begin to rise statistically significantly. This temperature coincides with the 89th percentile of the maximum daily temperature series for the summer months (May–September) across the period considered.

If, however, the mortality residuals are shown by reference to the minimum rather than the maximum daily temperature, a value of 18 °C is obtained, corresponding to the 91st percentile of the minimum temperature series of the summer months. This percentile is above the percentile observed for the maximum temperature (p89), which indicates that the maximum daily temperature is more appropriate for activating prevention plans since its percentile is lower.

Although using one temperature or another for the purpose of defining a hot day (i.e. one where heat-related mortality happens) in Antwerp may be of no consequence statistically speaking, from an epidemiological standpoint the effect of temperature on mortality is linked in greater measure to high maximum than to high minimum temperatures, which fundamentally give rise to discomfort and are not a direct cause of mortality (Diaz et al., 2015, Havenith, 2002). Therefore, in this study the threshold temperature indicator to define a hot day is set as the maximum daily temperature.

### 3.2 Impact of heat on mortality and cardiorespiratory emergency hospital admissions 2009–2013

Across the period 2009–2013, there were 82 days on which the maximum daily temperature rose above 26 °C, an annual average of 16.4 hot days. Heat-related mortality in this period totalled 67 persons (95% CI: 19–117), which translates as an annual average heat-related mortality of 13.4 persons (95% CI: 3.8–23.4), i.e. for each day on which there is a daily maximum temperature above 26 °C, there is a heat-related mortality of 0.82 persons (95% CI: 0.23–1.43).

Moreover, in Antwerp, heat was associated with daily mortality at lag 1. This is in line with previous results on the short-term impact of heat on mortality. The *RR* was 1.028 (95% CI: 1.008–1.049); and the *AR* was 2.7%, slightly lower than that reported elsewhere for places with similar mortality threshold percentiles (Carmona et al., 2016b). The *RR*s and *AR*s are shown below in Table 1.

**Table 1:** Relative risks (*RR*s) and attributable risks (*AR*s) for each degree that *T*_max_ exceeds 26 °C and for every 10 μg/m^3^ increase in *PM*_10_ concentrations.

Antwerp 2009–2013 Summer months(May–September)	Variable	*RR* (95% CI)	*AR* (95% CI)
Organic mortality	*T*_heat_ (lag 1)	1.028 (1.008–1.049)	2.7 (0.76–4.70)
	PM_10_ (lag 0)	1.044 (1.016–1.073)	4.3 (1.60–6.84)
Cardiorespiratory hospital admissions	PM_10_ (lag 0)	1.090 (1.045–1.138)	8.3 (4.27–12.13)

PM_10_ was observed to have an effect on organic-cause mortality and cardiorespiratory admissions, in both cases at lag 0, with an impact of 4.3% in the case of mortality and an impact of 8.3% in the case of admissions for every increase of 10 μg/m^3^. No effect of heat was observed in the case of hospital admissions due to cardiorespiratory causes.

### 3.3 Projected impact of heat on mortality 2026–2045 and 2081–2100

The impact of both heat and cold on populations' health is known to change over time, not only in terms of threshold temperatures, but also in terms of impacts as measured by *RR*s and *AR*s (Diaz et al., 2015, Miron et al., 2015, Roldan et al., 2016). This is a consequence of progressive adaptation to heat by the population (Bobb et al., 2014) and the ensuing prevention plans (Diaz et al., 2015). Modelling this variation across time is difficult, however: the retrospective time series has to be sufficiently long for the variations to be detected and modelled; and modelling the effect of prevention plans under a changing climate and population further complicates the process.

When confronted by this difficulty, it is common to assume for simplicity that both heat-related mortality threshold temperature and its corresponding impacts remain constant over time (Wu et al., 2014). The alternative is to assume that there *is* a process of acclimatization and that this translates as the fact that the cold- or heat-related threshold temperature has indeed varied across time. This can be achieved by assuming that the percentile of the series to which the threshold temperature corresponds will remain constant, and that, since temperatures are going to vary in the coming decades, the temperatures to which such percentiles correspond will also vary.

In this case, both possibilities are considered to determine, on the basis of the temperature series furnished for the periods 2026–2045 and 2081–2100 under RCP8.5 in Antwerp, the future impact of and mortality attributable to heat over this time horizon. In addition, mortality will be analysed for the entire period, and its impact will be analysed by reference to different intermediate time periods. Therefore, two hypotheses are considered in this study, with the respective assumptions being:

1. that the heat-related mortality threshold temperature, corresponding to a maximum daily temperature of 26 °C (89th percentile of the maximum daily temperatures for the months of May to September), is not going to vary across time, i.e. there is no heat-adaptation process; and,
2. that there *is* a heat-acclimatization process, which means that the threshold temperature may vary over the course of the different periods, with the 89th percentile being the element that remains constant throughout.

In all cases, the *AR* has been assumed to remain constant and equal to that calculated for the reference period 2009–2013, i.e. *AR*: 2.7% (0.76–4.70).

#### 3.3.1 Projected mortality under a no acclimatization scenario

With reference to the established threshold under this scenario (i.e. a maximum daily temperature of 26 °C), there would be 535 heatwave days in Antwerp during the period 2026–2045 under RCP8.5, with 26.7 hot days/year versus the current figure of 16.4 days, a 62.8% increase. Attributable mortality across this period would be 631 persons, with a mean heat-related mortality of 32 persons per year. This value is more than double that calculated for the retrospective period 2009–2013.

If the analysis is repeated by dividing the complete period of 20 years into four intermediate periods of 5 years each (as with the retrospective period 2009–2013), a general increase is seen in the number of hot days each year, rising from 24.6 per year in the period 2026–2030 to 30 in the last period (see Table 2). There is also an increase in annual heat-related mortality in each period. In the last period, 2041–2045, this mortality is about 39 persons per year, tripling that in the period 2009–2013.

**Table 2:** Heat - attributable mortality in the period 2026–2045, assuming a constant threshold temperature of 26 °C in different intermediate 5-year periods.

Period	Hot days	Organic attributable mortality	Daily organic attributable mortality	Organic attributable mortality (every year)
2026–2030	123	124.88 (35.15–217.39)	1.02 (0.29–1.77)	24.98 (7.03–43.48)
2031–2035	154	184.16 (51.84–320.57)	1.20 (0.34–2.08)	36.83 (10.37–64.11)
2036–2040	108	128.07 (36.05–222.93)	1.19 (0.33–2.06)	25.61 (7.21–44.59)
2041–2045	150	193.94 (54.59–337.60)	1.29 (0.36–2.25)	38.79 (10.92–67.52)

In turn, during the period 2081–2100 there would be 1075 days with a maximum daily temperature above 26 °C, i.e. heat-wave days, with 35.8 hot days/year. Attributable mortality across this period would be 1710 persons, with a mean heat-related mortality of 86 persons per year. This value is 6.6 times that of the period 2009–2013, i.e. 13 persons. Table 3 shows the results by intermediate periods of 5 years each for 2081–2100. Under this scenario, the hot days are multiplied by 3.6 in the year 2100 and attributable mortality is multiplied by 7 in relation to the reference period 2009–2013.

**Table 3:** Heat - attributable mortality in the period 2081–2100, assuming a constant threshold temperature of 26 °C in different intermediate 5-year periods.

Period	Hot days	Organic attributable mortality	Daily organic attributable mortality	Organic attributable mortality (every year)
2081–2085	252	390.93 (110.04–680.50)	1.55 (0.44–2.70)	78.19 (22.01–136.10)
2086–2090	273	487.15 (137.12–848.00)	1.78 (0.50–3.11)	97.43 (27.42–169.60)
2091–2095	250	355.50 (100.07–618.84)	1.42 (0.40–2.48)	71.10 (20.01–123.77)
2096–2100	300	476.06 (134.00–828.70)	1.59 (0.45–2.76)	95.21 (26.80–165.74)

#### 3.3.2 Projected mortality under a heat acclimatization scenario

This scenario assumes that the percentile of the series to which the threshold temperature corresponds will remain constant, and that, since temperatures are going to vary in the coming decades, the temperatures to which such percentiles correspond will also vary. In this case, that percentile that remains constant throughout is the 89th percentile of the maximum daily temperatures for the months of May to September, as determined in the period 2009–2013. This scenario would be the most conservative from a population acclimatization standpoint, and the goal would be to ensure that the heat-related mortality threshold temperature did not exceed the temperatures established for these periods.

Except for the first period, attributable mortality remains practically the same as in the reference period 2009–2013, i.e. 67 (19–117), as seen in Table 4. The number of hot days per year is always the same, since the threshold temperature is based on a percentile. These results are in line with a heat-acclimatization process, the fact that there is no variation in attributable risk, and that variations are solely due to temperature, since mortality has also been kept constant in each year.

**Table 4:** Heat-related mortality by period, with the percentile corresponding to the mortality threshold temperature kept constant.

Period	*T*_max_ threshold (p89)	Organic attributable mortality	Daily organic attributable mortality	Organic attributable mortality (every year)
2026–2030	27.33 °C	69.89 (19.67–121.65)	0.83 (0.23–1.45)	13.98 (3.93–24.33)
2031–2035	28.08 °C	84.53 (23.79–147.15)	1.01 (0.28–1.75)	16.91 (4.76–29.43)
2036–2040	27.16 °C	81.68 (22.99–142.18)	0.97 (0.27–1.69)	16.34 (4.60–28.44)
2041–2045	27.85 °C	96.53 (27.17–168.03)	1.15 (0.32–2.00)	19.31 (5.43–33.61)

In the 2081–2100 period the threshold temperature in which the attributable mortality is maintained along the period is above 30 °C, as can be observed in Table 5. Mortality is slightly increased in comparison with both the reference (2009–2013) and previously modelled (2026–2045) periods.

**Table 5:** Heat-related mortality by period, with the percentile corresponding to the mortality threshold temperature kept constant. Period 2081–2100.

Period	Threshold (p96)	Organic attributable mortality	Daily organic attributable mortality	Organic attributable mortality (every year)
2081–2085	30.26 °C	86.37 (24.31–150.35)	1.03 (0.29–1.79)	17.27 (4.86–30.07)
2086–2090	31.09 °C	95.36 (26.84–165.99)	1.14 (0.32–1.98)	19.07 (5.37–33.20)
2091–2095	30.07 °C	90.40 (25.44–157.35)	1.08 (0.30–1.87)	18.08 (5.09–31.47)
2096–2100	30.83 °C	97.11 (27.34–169.05)	1.16 (0.32–2.01)	19.42 (5.47–33.81)

## 4 Discussion

The heat-related mortality threshold temperature, corresponding to a daily maximum of 26 °C (89th percentile of the summer months) indicates that the hot day phenomenon understood as days hot enough to cause mortality is currently relatively frequent in Antwerp, with a mean of 16.4 “hot days” per year. This hot day definition temperature is in line with the climatic characteristics to which the Antwerp population is subjected (Curriero et al., 2002, Kovats et al., 2006), characterized by mild summers. However, it is not climate factors alone that exert an influence: there are also other aspects such as the population pyramid, and the over-65 age group in particular (Alberdi et al., 1998, Diaz et al., 2015, Montero et al., 2012) which have a higher probability of bed confinement, inability for self care or lack of social contact (Bouchama et al., 2007), pre-existing chronic diseases (Hajat et al., 2005, Huynen et al., 2001) as well as socioeconomic factors, such as access to home insulation and air conditioning (Ballester et al., 2011), the existence of infrastructures adapted to heat (Vandentorren et al., 2006, Montero et al., 2012) and the presence or absence of effective heatwave prevention plans (Abrahamson et al., 2009).

Heatwave definition temperatures based on epidemiological studies, such as this one, have an important advantage over those based on an exclusively climatological method, since they consider the above population-linked factors (Montero et al., 2010). Moreover, from the stance of the operational efficiency of prevention plans, a proper definition of the plan activation temperature is crucial because, if the trigger temperature yielded by the climatological method is higher than that obtained epidemiologically, plans would not be implemented while heat-related mortality may already be occurring. In contrast, if the climatological percentile is below the figure obtained, this would imply activation of the prevention plan on days on which it was not required, with the ensuing economic cost (Carmona et al., 2017).

If the minimum rather than the maximum daily temperature is used as a heatwave-definition indicator, the threshold would be 18 °C (91st percentile). This heat threshold percentile is higher than that based on the maximum daily temperature indicator, something frequently observed (Diaz et al., 2015). The fact that this percentile is higher than that corresponding to the maximum temperature supports the choice of maximum daily temperature as the heatwave definition indicator. The reason, aside from biological considerations (Havenith, 2002), is that a lower percentile implies that the prevention plan will always be activated on days on which the epidemiological evidence suggests it would be needed. For instance, if the maximum daily temperature was chosen as a threshold indicator in the period considered, it would have been exceeded 84 times, for a total of 70 occasions if the minimum temperature was chosen. That is, during 14 days there would have been mortality attributable to heat without preventions plans activated. A cautious approach to prevention would suggest using maximum daily temperature as an indicator to define a heatwave, an approach followed in several relevant studies (Basagaña et al., 2011, Tan et al., 2010, Xu et al., 2016, Yang et al., 2013).

It should be noted that the period 2009–2013 (the years for which we had outcome data) did not include any very hot summers. Hence, the percentile value may not very representative for the long-term climate (for which typically 20–30 years are considered).

The fact that the association between high temperatures and mortality is established at lag 1, i.e. a very short-term effect, is in line both with the biological mechanisms implicated, namely the quickness of onset and lethality of cardiovascular diseases in the elderly (Havenith, 2002), and the results yielded by other studies (Alberdi et al., 1998, Diaz et al., 2015).

Unfortunately, we could not perform the analysis separately for cardiovascular and respiratory hospital admissions because we only had aggregated data for both sets of causes. This could indeed explain the lack of an effect of heat on the hospital admissions in our analysis. Notwithstanding, the failure to detect an association between high temperatures and hospital admissions due to cardiorespiratory causes could also be explained by differences in the pattern of relationship between heat-related hospital admissions and mortality (Kovats et al., 2004, Linares and Diaz, 2008, Mastrangelo et al., 2006). Heat-related cardiovascular diseases tend to deteriorate so rapidly that patients die before being admitted to hospital. This occurs for mortality due to circulatory causes but not for mortality due to respiratory causes, which tend to display a longer-term progression (Alberdi et al., 1998). In our case, both the fact that circulatory- and respiratory-cause admissions were combined and that the number of daily emergency admissions due to circulatory causes is generally higher than that due to respiratory causes, may account for this lack of association.

The *RR* and *AR* values obtained for the city of Antwerp are slightly lower than the corresponding values for this percentile. For instance, taking areas with similar percentiles from a recent study (Diaz et al., 2015), the corresponding *AR*s would average 7%, much higher than the 2.7% found for Antwerp. As mentioned above, factors other than climate may modify this impact of heat on daily mortality (Montero et al., 2012).

The projected doubling in the number of hot days from the reference period (2009–2013) to the end of the first future period (2045) over this period is in line with other studies (Roldan et al., 2016). Evidently, this increase in hot days is compatible with IPCC predictions (IPCC, 2013), which indicate that heat waves will become increasingly frequent in Europe. From the point of view of the health impact, heat-related mortality increases, particularly under the no acclimatization scenario, are also consistent with recent studies (Martinez et al., 2016).

While valid as a reference point, the no acclimatization scenario - which also assumes no adaptation - is unrealistic. As mentioned, demographic and socioeconomic factors might do, and will likely continue to influence the trend in mortality-inducing temperatures (Miron et al., 2008). Furthermore, the impact of heat on mortality, far from remaining constant, is changing over time with a decreasing trend (Schifano et al., 2012), particularly for cardiovascular mortality (Ha and Kim, 2013), while in the case of respiratory-cause mortality the effect remains practically constant (Miron et al., 2015). This trend seems to be linked to improvements in health services, socioeconomic shifts, the provision of infrastructures for better living conditions and the acclimatisation of the population to heat (Konkel, 2014).

Modelling the heat-acclimatization process through a rise in the heatwave threshold temperature associated to a constant percentile of future temperature series under climate change may serve to test whether the adaptive processes currently being implemented are adequate to the task in a changing climate. In the case of Antwerp, the heat-related mortality threshold temperature across the period 2041–2045 should not exceed the present temperature of 26 °C by > 2 °C and for period 2096–2100 should maintain about 5 °C above the present temperature. Furthermore, it should also be borne in mind that annual mortality will gradually rise over time, resulting in an increased heat-related mortality as well.

Mention should be made of a number of possible limitations and their ensuing biases. Firstly, with reference to the quality and consistency of the data analysed, there may be misclassification of the cause of mortality or admission, or errors related to the lack of data on environmental variables. Secondly, with respect to methodological limitations, essentially two should be highlighted: the shortcomings inherent in a statistical method that works with a high number of variables at a 95% confidence level. The fact that this is an ecological study means that inferences cannot be made at an individual level, since these would be exposed to the appearance of the ecological fallacy due to the use of aggregate data. It is only possible to show the existence of a statistical association between the variables analysed. Factors favouring the existence of a real, as opposed to a spurious-relationship, are the high correlation index, increasing dose-response relationship, absence of temporal ambiguity, biological plausibility of the effect shown in clinical studies, and consistency of results obtained by previous studies. Furthermore, in the case of estimates of the impact of temperatures on mortality, various hypotheses were postulated, including the assumption of mortality being constant over time, the related attributable risk remaining similarly constant, as well as the mortality threshold temperature associated with the extreme temperatures. In addition, there are great uncertainties inherent to population projections, including migration, age structure and mortality rates, albeit these may arguably be more acceptable than the obvious alternative of assuming a constant population or a simplified model. Also, our models did not account for potential changes in air pollution and urban heat related to changes in traffic policy (e.g. the establishment of a low emission zone) and in urban green (e.g. replacement of large old city trees by new trees).

There are various ways to design and implement effective prevention against the health impacts of heat. A common template to organize such prevention is a Heat-health action plan (WHO, 2008) in which a set or core elements and agencies hierarchically act upon and respond to heat warnings. Many prevention plans were formulated following the European heatwave in 2003. Currently various plans and additional activities are in place to tackle the issues related to heat in the Antwerp area. The Belgian National Heatwave plan defines a heatwave as a period of at least three consecutive days where the mean maximum temperature exceeds 30 °C and the mean minimum temperature exceeds 18 °C. The plan contains two warning levels: Level one is activated when two days with temperatures exceeding those mentioned have occurred. Level two is activated when the definition of a heatwave is fulfilled. Warnings are prepared during level one. The effectiveness of this action plan has been examined and improvements considered (van Loenhout et al., 2016). In addition to the National plan, the Flemish Region authorities have designed and are in the process of implementing a regional heat health action plan. In the proposed Flemish heat action plan the level two warning level is activated when a cumulative indicator of temperature, *T*_cumul_, equals or exceeds 17 °C. *T*_cumul_ is calculated as the sum of all degrees centigrade higher than 25 °C of the expected maximum temperature in a time window of five days. This method would allow activation of heat action plans three days in advance of an expected heatwave. The level two warning level stays active until the maximum temperature expected is lower than 25 °C. A sensitivity analysis of this new threshold showed that the new method resulted in longer activation periods of warning level two compared to the heat plan active at present, but the frequency of activation of level two did not differ between the two methods (Bustos-Sierra et al., 2016). Moreover, the municipality of Antwerp itself plans and runs every year a number of preventive activities. The results of this study can help calibrate these preventive efforts, and put a long term perspective on heat-health prevention in a changing climate.

## 5 Conclusions

Heat is causing significant excess summer mortality in Antwerp, and this mortality may increase in the future in the absence of adequate prevention, including the adaptation of such prevention to a changing climate. Heat-health action plans and their activation should always be based on the best available evidence, and specifically on activation thresholds based on epidemiological data and analysis. The acclimatisation of the population should be accounted for, as should realistic projections of demographic and mortality trends. More research is needed to fulfil the local evidence gap for effective prevention against heat, particularly in mid-sized urban agglomerations.

## List of abbreviations

RCP: Representative Concentration Pathway
IPCC: Intergovernmental Panel on Climate Change
ECMWF: European Centre for Medium-Range Weather Forecasts
GCM: global climate models
ICD: International Classification of Diseases
WPP: World Population Prospects
ARIMA: autoregressive integrated moving average
GLM: generalized linear model
RR: relative risk
AR: attributable risk

## Ethics approval and consent to participate

Not applicable.

## Consent for publication

Not applicable.

## Competing interests

The authors have no competing interests, financial or otherwise.

## Funding

The research leading to these results has received funding from the European Community's Seventh Framework Programme under Grant Agreement No. 308497 (Project RAMSES). The funding body (European Commission) did not play any role in the design of the study and collection, analysis, interpretation of data or in writing the manuscript. Additional funding was received from ISCIII (Project FIS ENPY 1133/16).

## Authors' contributions

GSM designed and coordinated the study, and drafted this article; JD coordinated and supervised the epidemiological analysis, which was carried out by CL, RC and CO. HH and DL collaboratively carried out the urban climate and climate change modelling, with the supervision and collaboration of KDR. AVN, RA and VK provided inputs and context for the discussion and introduction sections. MBND collected and analysed population data, formatted the draft and references, and managed the peer review coordination among authors.
